# How varying parameters impact insecticide resistance bioassay: An example on the worldwide invasive pest *Drosophila suzukii*

**DOI:** 10.1371/journal.pone.0247756

**Published:** 2021-03-05

**Authors:** Lucile Blouquy, Claire Mottet, Jérôme Olivares, Christophe Plantamp, Myriam Siegwart, Benoit Barrès

**Affiliations:** 1 Université de Lyon, Anses, INRAE, USC CASPER, Lyon, France; 2 PSH - Unité de recherche Plantes et Systèmes de Culture Horticoles, INRAE, Avignon, France; Northwest A&F University, CHINA

## Abstract

Monitoring pesticide resistance is essential for effective and sustainable agricultural practices. Bioassays are the basis for pesticide-resistance testing, but devising a reliable and reproducible method can be challenging because these tests are carried out on living organisms. Here, we investigated five critical parameters and how they affected the evaluation of resistance to the organophosphate phosmet or the pyrethroid lambda-cyhalothrin using a tarsal-contact protocol on *Drosophila suzukii*, a worldwide invasive pest. Three of the parameters were related to insect biology: (i) sex, (ii) age of the imago (adult stage) and (iii) genetic diversity of the tested population. The two remaining parameters were linked to the experimental setup: (iv) the number of individuals tested per dose and (v) the duration of exposure to the active ingredient. Results showed that response to insecticide differed depending on sex, males being twice as susceptible to phosmet as females. Age principally affected young females’ susceptibility to phosmet, because 0–24 hour-old flies were twice as susceptible as 24–48 hour-old and 72–96 hour-old females. Genetic diversity had no observable effect on resistance levels. The precision and accuracy of the median lethal dose (LD_50_) were greatly affected by the number of individuals tested per dose with a threshold effect. Finally, optimal duration of exposure to the active ingredient was 24 h, as we found an underestimation of mortality when assessed between 1 and 5 h after exposure to lambda-cyhalothrin. None of the main known point mutations on the *para* sodium channel gene associated with a knockdown effect were observed. Our study demonstrates the importance of calibrating the various parameters of a bioassay to develop a reliable method. It also provides a valuable and transferable protocol for monitoring *D*. *suzukii* resistance worldwide.

## Introduction

Bioassays are considered as the gold standard for pesticide resistance testing. They can detect new resistances and can assess resistance in an integrative manner, whether the underlying mechanism is known or unknown [1]. With the growing ambition to employ sustainable practices of pest control, monitoring pesticide resistance is becoming the key to implementing efficient, integrated pest management strategies. Accurate and reliable phenotypic data, in addition to molecular data, are also essential to address numerous theoretical evolutionary questions linked to pesticide resistance (see [2] for examples with insecticides). As in most experiments involving living material, the output of pesticide resistance bioassays can be affected by many parameters, depending on the type of bioassay used and the study species.

When dealing with a new species/active substance pair, dose-response analyses are the most frequently used approach. The standard procedure consists in exposing insects to a range of doses of the active substance of interest and in counting the number of dead individuals for each dose. Using regression methods, it is then possible to determine various parameters of interest, and in particular the median lethal dose (LD_50_), the dose that kills 50% of the exposed individuals. Results of bioassays may be affected by experimental conditions and design [3] and also by the biological characteristics of the insect species [4–8]. For each pest, it is necessary to develop, or at least to adapt, a standard experimental protocol to obtain reliable and repeatable results that will allow comparisons of pesticide susceptibility over space and time.

In this study, we used the spotted wing drosophila *Drosophila suzukii* (Matsumura) for a case study. It is a harmful pest that severely affects fruit production and causes severe economic losses [9–11] by altering berries and other soft-skinned fruits. *D*. *suzukii*, native to Asia, is also known as a worldwide invasive species [12, 13] and was first introduced in North America and Europe in 2008 before spreading throughout the globe [14–18]. The main control strategies for *D*. *suzukii* rely on chemical insecticides [19, 20] and marginally on alternative control methods [21–35]. Its dispersal and reproductive abilities make it a species with high evolutionary potential [17, 36], and it may be prone to evolve insecticide resistance [37]. Moreover, other species of the *Drosophila* genus have already demonstrated their ability to resist insecticides [38–41]. All these reasons call for a careful and large-scale plan to monitor insecticide resistance in this species, but such monitoring plans require reliable and standardized methods. To date, studies have evaluated pesticide efficiency or resistance in *D*. *suzukii* populations using various protocols [4, 20, 37, 42–53], without extensive investigations on the reliability of the methods used.

Because most insecticides targeting *D*. *suzukii* belong to the group of contact insecticides, we chose to use tarsal-contact bioassays. This type of bioassay is easy to set up and relatively inexpensive, facilitating its use worldwide. Organophosphates and pyrethroids have been the most effective and the most used insecticides in the field in France [42–44, 47, 49]; we therefore focused our experiments on two active substances of these families commonly used in Europe against *D*. *suzukii*: the organophosphate phosmet and the pyrethroid lambda-cyhalothrin. The formulated products act via contact, ingestion and inhalation for phosmet and via contact and ingestion for lambda-cyhalothrin.

The main aim of this study was to illustrate and to investigate how various biological and technical parameters can affect the accuracy and reliability of a pesticide resistance bioassay. The key biological parameters we explored were (i) sex, (ii) age of the imago (adult stage) and (iii) genetic diversity of the tested population. Two methodological parameters were also tested: (iv) number of individuals used to evaluate the level of insecticide resistance and (v) duration of exposure to the active substance. One interesting and valuable output of this work is a reliable, accurate and standardized tarsal-contact bioassay to test insecticide resistance. Our work highlights the essential factors to control in the development of reliable bioassays on any other pest species.

## Materials and methods

### Sample collection and preparation

The Ste-Foy population used in the experiments was originally established from approximately 20 *D*. *suzukii* females, collected from southeastern France in an urban area on raspberries (Sainte-Foy-les-Lyon, France) in May 2012 by Roland Allemand (Biometry and Evolutionary Biology Laboratory, CNRS–University of Lyon). The population was then mass-reared and maintained in standard drosophila vials (Ø 25 mm x h 95 mm) containing ~10 ml of a standard food media composed of 10 g of agar diluted in 1 l of water, 60 g of glucose, 30 g of saccharose, 80 g of malted yeast, 20 g of yeast extract, 20 g of peptones, 0.5 g of magnesium sulfate, 0.5 g of calcium chloride and 1 g of nipagin, pre-diluted in 10 ml of ethanol. They were maintained in a climatic chamber at 23±1°C, under a relative humidity of approximately 70% and a 16:8 h light-dark cycle. Temperature was strictly controlled because it can influence *D*. *suzukii* susceptibility to insecticides [54]. Then, 25–30 males and females per vial were left to mate and oviposit for four to seven days before being removed from the rearing vials. After two weeks, the newly emerged adults (imagoes) were isolated and transferred to a new vial to start a new generation. To maintain the Ste-Foy population, each generation consisted of approximately 20 vials and the emerged adults of all the vials were mixed together to maximize genetic variability before distributing them into new vials.

A low genetic diversity population, SF-IsoA, was generated from the Ste-Foy population by inbreeding the flies for three generations: each generation, a single brother and virgin sister pair were isolated and left to reproduce in a new vial. The SF-IsoA population was then maintained in the same conditions as the Ste-Foy population.

### Tarsal-contact bioassays

All the experiments were conducted using a common framework for the bioassays. Based on this framework, the five parameters of the protocol were modified to test for their effect on the assessed pesticide resistance. The tarsal-contact bioassay consists in exposing fly distal part of the legs (tarsi) to insecticide for a pre-determined time before assessing mortality. We used 20 ml scintillation glass vials (Ø 28 mm x h 61 mm), which have previously been used on *D*. *suzukii* with good results for resistance monitoring [48, 52]. A volume of 500 μl of the insecticide solution at the desired concentration (dissolved in acetone) was deposited on the walls of the vials. The insecticide solution was uniformly distributed with acetone evaporation by rolling the vials at room temperature for 1 h, on a hot-dog roller. Seven doses of the active substance were tested, including a control with acetone only. The range of concentrations differed for the two insecticides and depended on what concentrations allowed a dose-response curve ranging from 0 to 100% mortality. For the organophosphate phosmet (95% of purity), an acetylcholinesterase inhibitor and obtained from Gowan, the chosen range of concentrations was 0, 4.7, 9.4, 18.9, 37.7, 75.5 and 150.9 mg/l. Lambda-cyhalothrin (92.8% of purity) is a pyrethroid that targets the sodium channel and was furnished by Syngenta France SAS. The selected concentration range was adjusted according to the bioassay and consisted in 7 doses chosen from the following concentrations: 0, 0.01, 0.05, 0.1, 0.25, 0.5, 1, 3 and 5 mg/l. Because contact time lasted up to 24 h, 100 μl of an agarose-sucrose mixture (5% sucrose, 8 g/l agarose) was provided in each vial to prevent death by starvation. For a bioassay, one to four vials for the same dose were used, depending on the number of flies tested. The number of *D*. *suzukii* flies of a uniform age class (the standard being 24 to 48 hours old in this study) from a single population ranged from 3 to 32 by vial. Males and females were not separated prior to the bioassay to avoid anesthesia (necessary to determine the sex of the flies). The vials were plugged with thin netting held in place by perforated caps to prevent flies from escaping and still allow good ventilation. The vials were maintained in a climatic chamber during the bioassay at 20±1°C, under a relative humidity of approximately 75% and a 16:8 h light-dark cycle. After a certain duration of exposure (the standard being 24 h), the vials were briefly shaken and the numbers of dead, moribund and live flies were assessed. Individuals that could not remain on their legs and those that showed unusual behavior (*i*.*e*. uncertain or irregular flight, twitching legs and/or uneven movements) were considered as moribund. Immobile adult flies were considered dead. The sex of the flies for the different categories was determined during this mortality assessment by checking for the presence of black spots on the wings which characterizes males. The susceptibility of a population to the chemical was assessed by calculating the LD_50_ value (see Statistical analysis section below).

This protocol was used for conducting five experiments with some variation regarding the *D*. *suzukii* individuals or the conditions of the bioassay (see Table 1). For each experiment, several bioassays were performed and each bioassay was done on a different date. Within a bioassay, multiple vials per dose could be used in order to have a limited number of flies per vial (up to 32). For *Experiment 1* (24 bioassays), the test aimed to compare the susceptibility to phosmet according to **sex** at 24 h of exposure on flies from Ste-foy population aged of 24 to 48 h. In *Experiment 2* (26 bioassays), we explored the influence of **age class** on susceptibility of Ste-Foy population to 24h of exposition to phosmet by testing males and females of three different age classes: 0 to 24 h, 24 to 48 h and 72 to 96 h. *Experiment 3* (5 bioassays) consisted in observing the impact of the **genetic diversity** of a population on its resistance to phosmet after 24h of exposure. To do so, the reference population Ste-Foy was compared to the inbred SF-IsoA population and flies were 24 to 48 h old. The respective levels of genetic diversity of the two populations were assessed using molecular tools (see below). *Experiment 4* (24 bioassays) investigated the impact of the **number of flies** tested per bioassay (mean number of flies per dose per bioassay ranging from 6 to 37 for the females and from 9 to 35 for the males, see Table 2) on the accuracy of the LD_50_ estimates after 24 h of exposure of 24 to 48 h old flies from Ste-Foy to phosmet. Finally, *Experiment 5* (3 bioassays) explored the effect of the **duration exposure** to pyrethroid insecticide (lambda-cyhalothrin) on the evaluation of susceptibility of Ste-Foy population. Unlike organophosphate insecticides, pyrethroid can potentially induce a knockdown effect (due to several mutations in the sodium channel molecular target), which may cause an erroneous mortality assessment depending on the time of observation after initial exposure to the chemical. That is why we conducted the bioassays with lambda-cyhalothrin in this *Experiment 5*. Mortality was assessed repeatedly on the same vials at 10 different times: 1, 2, 3, 4, 5, 20, 21, 22, 23 and 24 h after initial exposure.

**Table 1:** Parameters tested in the five experiments.

Colonne1	Tested parameters	Insecticide	Population	Age of the flies	Exposure duration to insecticide	Number of bioassays
***Experiment 1***	Sex	Phosmet	Ste Foy	24-48h	24h	24
***Experiment 2***	Age	Phosmet	Ste Foy	0-24h; 24-48h; 72-96h	24h	26
***Experiment 3***	Genetic diversity	Phosmet	Ste Foy; SF-IsoA	24-48h	24h	5
***Experiment 4***	Insect number per dose	Phosmet	Ste Foy	24-48h	24h	24
***Experiment 5***	Duration of insecticide exposure	Lambda-cyhalothrin	Ste Foy	24-48h	1; 2; 3; 4; 5; 20; 21; 22; 23; 24h	3

**Table 2:** Characteristics of the study’s bioassays. For each of the bioassays, the date of performance, the inclusion in the different experiments, the mean number of flies per dose (and per sex) and the total number of individuals used are indicated.

						Mean number of flies per dose			
Date of the bioassay	*Experiment 1*	*Experiment 2*	*Experiment 3*	*Experiment 4*	*Experiment 5*	♀	♂	♀♂	Total number of flies per bioassay
25/05/2016	X	X		X		20	14	34	238
01/06/2016	X	X		X		23	20	43	299
08/06/2016	X	X		X		15	16	31	216
13/06/2016		X				9	7	16	111
14/06/2016		X				22	21	42	296
16/06/2016		X				21	15	36	253
20/06/2016		X				9	7	16	112
22/06/2016	X	X		X		22	21	44	305
23/06/2016	X	X		X		32	29	61	424
27/06/2016		X				11	7	18	128
28/06/2016		X				13	13	26	184
30/06/2016	X	X		X		18	15	33	231
04/07/2016		X				13	12	25	173
05/07/2016		X				12	9	22	152
07/07/2016	X	X		X		18	14	32	225
11/07/2016		X				11	8	19	134
12/07/2016		X				6	4	10	72
21/07/2016	X	X		X		13	9	22	156
25/07/2016		X				13	8	22	152
01/09/2016	X	X		X		13	10	23	163
08/09/2016	X	X		X		13	14	27	190
15/09/2016	X	X		X		20	17	36	254
22/09/2016	X	X		X		23	18	41	288
13/10/2016	X	X		X		10	10	20	140
20/10/2016	X	X		X		10	10	20	140
27/10/2016	X	X		X		12	11	24	167
01/03/2017	X		X	X		17	17	34	238
15/03/2017	X		X	X		18	15	33	233
23/03/2017	X		X	X		14	15	29	205
29/03/2017	X			X		18	12	30	209
05/04/2017	X		X	X		21	16	37	261
06/04/2017	X		X	X		24	22	47	326
04/05/2017	X			X		37	29	66	463
11/05/2017	X			X		36	30	66	460
17/05/2017	X			X		34	35	69	482
09/09/2020					X	37	24	61	428
30/09/2020					X	23	13	35	248
01/10/2020					X	38	28	66	459

♀–Female flies

♂–male flies of Ste-Foy or SF-IsoA population of *Drosophila suzukii*.

### Assessing the neutral and adaptive genetic diversity of the populations

#### DNA extraction

Adults (aged from 24 to 72 h old) were picked from the rearing stock in order to assess the genetic diversity of Ste-Foy and SF-IsoA populations. Flies were killed directly in 70% ethanol and the total DNA was extracted from the whole body within a week. DNA from 44 individual *D*. *suzukii* samples (30 females, 14 males) from each population (Ste-Foy and SF-IsoA) was extracted following a modified method based on Walsh *et al*. (1991) [55]. A volume of 100 μl of a 10% Chelex 100 solution (Bio-Rad) and 3% of 10 mg/ml proteinase K (Eurobio) was added to each sample. Then, each sample was crushed using 2 mm steel beads on a 1600 MiniG tissue homogenizer (Spex® SamplePrep) at 1500 strokes/min for 5 sec, in a 96-well format PCR plate. Tissues were digested for 14 h at 56°C with a Mastercycler thermocycler (Eppendorf) with a final temperature step of 30 min at 98°C, the supernatant was used as DNA template for PCR reaction.

#### Microsatellite genotyping

The genetic diversity of the two studied populations was assessed using microsatellite markers. We used a slightly modified nested PCR approach described by Schuelke [56] coupled with a selection of 13 microsatellite markers developed by Fraimout *et al*. [57] (S1 Table). A forward specific primer was conjugated with a 5’-GTTGTAAAACGACGGCCAGT-3’ M13-tail at its 5’ end, the same labeled universal M13-tail was used for fluorescence detection. We used a 1:10 ratio with 1 unit tailed forward specific primer to 10 units labeled M13 tails and 10 specific reverse primers. PCR amplifications were carried out on a Mastercycler thermocycler (Eppendorf) in a 12 μl reaction volume containing 1X GoTaq® Flexi Buffer, 1.5 mM MgCl_2_, 0.1 mg/ml bovine serum albumin (BSA), 200 μM of each dNTP, 0.4 μM of the labelled M13 tail, 0.4 μM of the specific reverse primer, 0.04 μM of M13-tailed specific forward primer, 1 unit of GoTaq® Flexi DNA Polymerase (Promega) and 2 μl of DNA template. The PCR conditions were: 3 min at 95°C followed by 30 cycles at 95°C for 30 sec, 57°C for 45 sec, 72°C for 45 sec and 10 cycles at 95°C for 30 sec, 54°C for 30 sec, 72°C for 45 sec with a final extension step at 72°C for 20 min.

Labeled PCR products were pool-plexed (up to 7 loci) by using 2–8 μl of each PCR in 50 μl of H_2_O, according to their 5’ end-labeled dyes (dilution 1:8 for Tamra, 1:16 for Hex and Atto-565 and 1:32 for 6-Fam Dyes). A volume of 2 μl of the diluted PCR mixture, 7.8 μl of HiDi formamide, and 0.2 μl GeneScan™ 600 LIZ® size standard (Applied Biosystems) were injected in an ABI 3730xl DNA Analyzer (Applied Biosystems) using POP7 polymer. These genotyping runs were analyzed using GeneMapper® V4.1 Analysis Software (Applied Biosystems).

#### Checking for the *kdr* mutation

Knockdown resistance to pyrethroids is known to be induced by some mutations in its molecular target, the voltage-gated sodium channel. To assess the presence of the main *kdr* mutations that lead to the L1014F substitution [58], we adapted a PCR-RFLP protocol from Franck *et al*. [59]. Twenty adults (aged from 24 to 72 h old) were randomly picked from the rearing stock of Ste-Foy population as well as 20 flies from the same population that survived an exposition of 24 h at 0.25 mg/l of lambda-cyhalothrin (from *Experiment 5*) and their DNA was extracted as described above. A 371 bp PCR fragment was amplified on a Mastercycler thermocycler (Eppendorf) in a 12 μl reaction volume containing 1X GoTaq® Flexi Buffer, 1.5 mM MgCl_2_, 0.1 mg/ml BSA, 200 μM of each dNTP, 0.4 μM of each primer (CKDR1 and Cgd2; see Table 3), 1 unit of GoTaq® Flexi DNA Polymerase (Promega) and 2 μl of DNA template. The PCR conditions were: 3 min at 95°C followed by 35 cycles at 95°C for 30 sec, 56°C for 45 sec, 72°C for 45 sec with a final extension step at 72°C for 20 min. The PCR products (5 μl) were digested at 37°C for 16 h with 2 units of *Mlu*CI endonuclease and 1X of NEB Buffer (New England Biolabs) in 10 μl of reaction volume. The digested products (4 μl) were separated on a 2% agarose gel at 100 V for 30 min using the RunOneTM Electrophoresis System (Embi Technology). The size of the fragments was estimated by comparison with a 100 bp DNA ladder (Promega). The L1014F mutation reveals a restriction site recognized by the *Mlu*CI endonuclease, which cuts the 371 bp PCR fragment into two fragments of respectively 123 bp and 248 bp. Susceptible genotypes remain undigested.

**Table 3:** Primers used to amplify and sequence the trans-membrane segments 4 to 6 of the domain II region of the voltage-gated sodium channel of *Drosophila suzukii*.

PRIMER NAME	SEQUENCE (5’→ 3’)
Ds-SKdr-F	TGGCCAACACTTAATTTACTC
Ds-seq-R	CAAGAAGAAGGGAATGCAC
CKDR1	CACAGCTTCATGATCGTGTTC
Cgd2	GCAAGGCTAAGAAAAGGTTAAG

Primers Ds-SKdr-F and Ds-Seq-R were designed specifically for this study. Primers CKDR1 and Cgd2, designed in our laboratory and used in previous studies [60, 61], were used as is given their perfect homologies.

#### Voltage-gated sodium channel gene sequencing

To verify the absence of the L1014F substitution and other secondary substitutions previously described in other insect species (i.e. M918T, L925I, T929I, L932F, C933A, I936V, G943A, Q945R, I1011M/V, N1013S and V1016G) [62–65], we relied on the complete genome of *D*. *suzukii* (GenBank assembly accession: GCA_000472105.1) focusing on scaffold023, positions 597.317 to 698.969, which correspond to the trans-membrane segments 4 to 6 of the domain II region of the voltage-gated sodium channel (S1 Fig). A 1564 bp PCR fragment was amplified for two individuals from Ste-Foy population (that survived an exposition of 24 h at 0.25 mg/l of lambda-cyhalothrin) on a Mastercycler thermocycler (Eppendorf) in a 30 μl reaction volume, containing: 1X GoTaq® Flexi Buffer, 1.5 mM MgCl_2_, 0.1 mg/ml BSA, 200 μM of each dNTP, 0.4 μM of each primer (Ds-SKdr-F and Cgd2) (see Table 1), 1 unit of GoTaq® Flexi DNA Polymerase (Promega) and 2 μl of DNA template. The PCR conditions were: 3 min at 95°C followed by 35 cycles at 95°C for 30 sec, 54°C for 45 sec, 72°C for 2 min with a final extension step at 72°C for 20 min. The PCR products (20 μl) were sequenced (Eurofins Genomics) using primers Ds-SKdr-F, Ds-Seq-R and CKDR1 (see Table 3). DNA sequences were manually aligned and analyzed using Bioedit software [66].

### Statistical analysis

Moribund and dead individuals were combined and considered as dead. Prior to data analyses, we kept only bioassays with a mortality rate in the control less than 15%. Survival data were analyzed in a non-linear regression framework using the ‘drc’ package [67] in r [68]. For all experiments, we assumed a binomial distribution of errors. For *Experiments 1*, *2* and *3*, survival data were fitted to the three-parameter log-normal model, which is equivalent to the classic probit model [69] with an additional parameter that takes into account the ‘natural’ mortality rate observed in the control of each categories. Due to the very low mortality rate for the different categories of *Experiment 4* and *Experiment 5* (<10%) as well as the sometimes limited number of individuals tested, survival data were fitted to the two-parameter log-normal model equivalent of the classic probit model [69]. For *Experiments 1*, *2 3* and *5*, the results of the bioassays were homogeneous and were therefore pooled together. The results of the bioassays included in *Experiment 4* were not pooled because the purpose of this experiment was to assess the effect of the mean number of individuals in the bioassay on the LD_50_ estimates. Models were fitted for the different categories separately within each experiment. The ‘drc’ package allows the estimation of LD_50_ and the associated 95% CI and standard errors. Difference in LD_50_ between males and females (*Experiment 1*) was tested directly using the ‘compParm’ function implemented in the ‘drc’ package. For the other experiments, the datasets were split into male and female subsets prior to the analysis. Pairwise comparisons of LD_50_ between the different age classes, the populations with different levels of genetic diversity and the different times of exposure were performed for each sex separately for *Experiments 2*, *3* and *5*, respectively, using the ‘compParm’ function. LD_50_ estimated on each bioassay of *Experiment 4* separately were compared with the LD_50_ estimated on the bioassays pooled by sex based on the overlapping of the 95% CI and the respective values. Additionally, for *Experiment 3*, two genetic diversity indices were computed for the Ste-Foy and the SF-IsoA populations. The number of alleles (*N*_a_) and gene diversity (*H*_e_) [70] were estimated for both populations and for all loci with genepop V4.2.2 [71]. The dataset and the code used for the different analyses, as well as for the production of the figures, have been deposited in an online repository (doi: 10.5281/zenodo.2842939).

## Results

### Effect of biological parameters on LD_50_ estimates: Sex, individual fly age and population genetic diversity

#### The influence of sex on insecticide susceptibility

**Fig 1:**
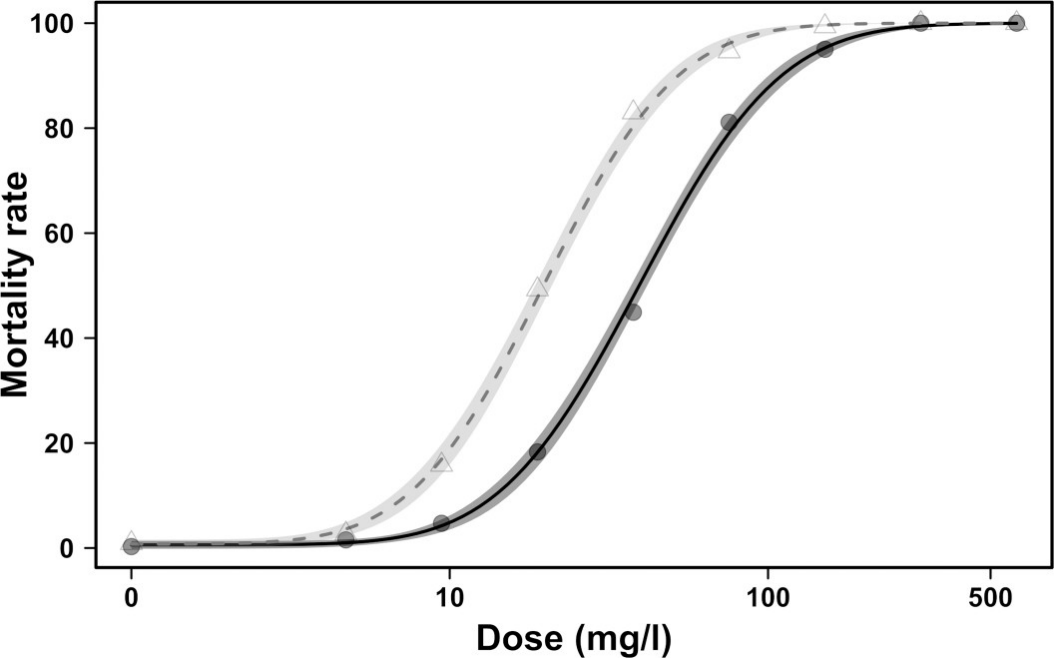
Effect of sex on LD_50_ in a *D*. *suzukii* population. Dose-response curves of 24 to 48 h old male (light gray triangles, shading and dashed line) and female (dark gray circles, shading and solid line) adults of *D*. *suzukii* after 24 h of tarsal exposure to phosmet. The 95% confidence intervals were derived from the dose-response model.

A total of 2745 females and 2341 males were tested in 24 bioassays. The LD_50_ values for phosmet were estimated at 39.6 (95% CI: 37.2–42.0) and 19.8 (95% CI: 18.6–21.1) mg/l for females and males, respectively (Fig 1). The two-fold higher resistance to phosmet of females compared with males was highly significant (relative female:male potency = 1.99, t-value = 11.3, p-value < 0.001).

#### The influence of fly age on insecticide susceptibility

**Fig 2:**
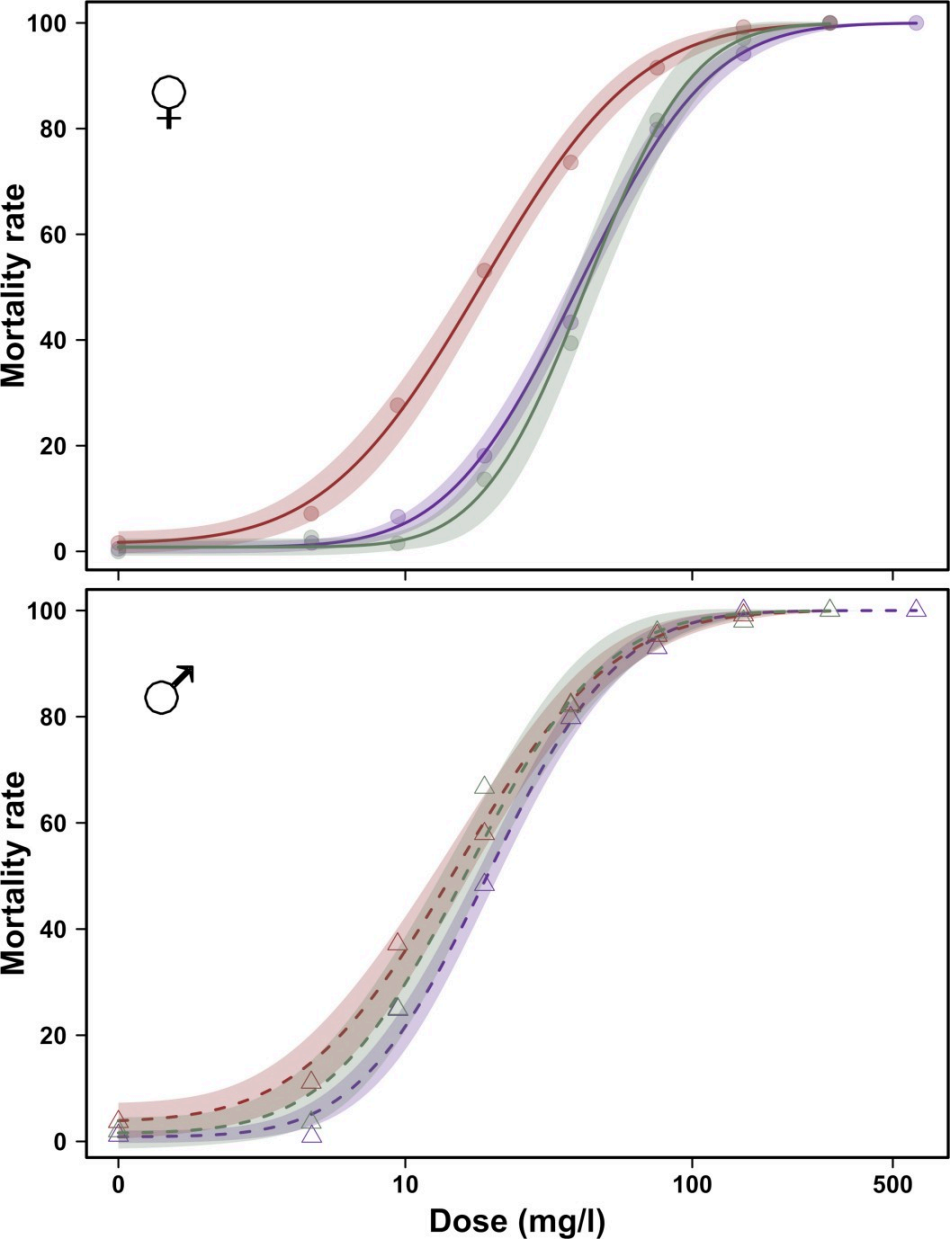
Effect of age class on LD_50_ in a *D*. *suzukii* population. Results obtained after 24 h tarsal exposure to phosmet for females (upper panel) and males (lower panel). Red, green and blue represent the 0–24 h, 24–48 h and 72–96 h age classes, respectively. Dose-response curves and 95% confidence intervals were derived from the dose-response model.

A significant effect was observed between the 0–24 h and 24–48 h age classes for males (relative potency 0–24 h:24–48 h = 0.78, t-value = -3.09, p-value = 0.002). No significant differences were observed between the other different age classes for male flies (relative potency 0–24 h:72–96 h = 0.93, t-value = -0.64, p-value = 0.523 and relative potency 24–48 h:72–96 h = 1.19, t-value = 1.51, p-value = 0.132) (Fig 2). The estimates of the LD_50_ values were very similar for the three age classes with 15.2 mg/l (95% CI: 12.9–17.5), 19.5 mg/l (95% CI: 17.7–21.2) and 16.4 mg/l (95% CI: 13.4–19.4), from youngest to oldest.

Unlike males, age seemed to involve a clear change in insecticide susceptibility for females (Fig 2). The two older age classes showed similar LD_50_ values with 40.3 mg/l (95% CI: 36.9–43.8) for the 24–48 h females and 42.9 mg/l (95% CI: 37.3–48.5) for the 72–96 h age class (relative potency 24–48 h:72–96 h = 0.94, t-value = -0.80, p-value = 0.421). The youngest females were twice as susceptible to phosmet, with a LD_50_ value of 18.6 mg/l (95% CI: 16.2–20.9), compared with the two other age classes (relative potency 0–24 h:24–48 h = 0.46, t-value = -14.9, p-value < 0.001 and relative potency 0–24 h:72–96 h = 0.43, t-value = -14.0, p-value < 0.001).

#### The influence of genetic diversity on insecticide susceptibility

**Fig 3:**
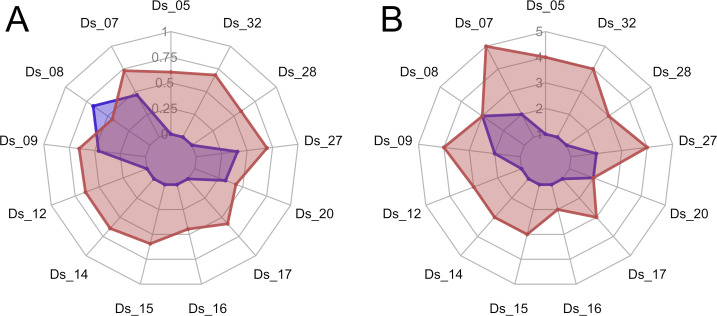
Diversity indices of the Ste Foy and SF-IsoA populations. Radar plot of (A) gene diversity (*H*_e_) and (B) the number of observed alleles (*N*_a_) at 13 microsatellite loci for the Ste-Foy (red) and SF-IsoA populations (blue).

Microsatellite genotyping confirmed that the genetic diversity of the SF-IsoA population was reduced compared with the Ste-Foy population at 13 loci (Fig 3 and S2 Table). The mean number of alleles (per microsatellite marker) and the mean gene diversity was higher in Ste-Foy (*N*_a_ = 3.31; *H*_e_ = 0.595) than in SF-IsoA (*N*_a_ = 1.46; *H*_e_ = 0.179), confirming that Ste-Foy and SF-IsoA populations are appropriate biological materials to test the influence of genetic diversity on resistance.

**Fig 4:**
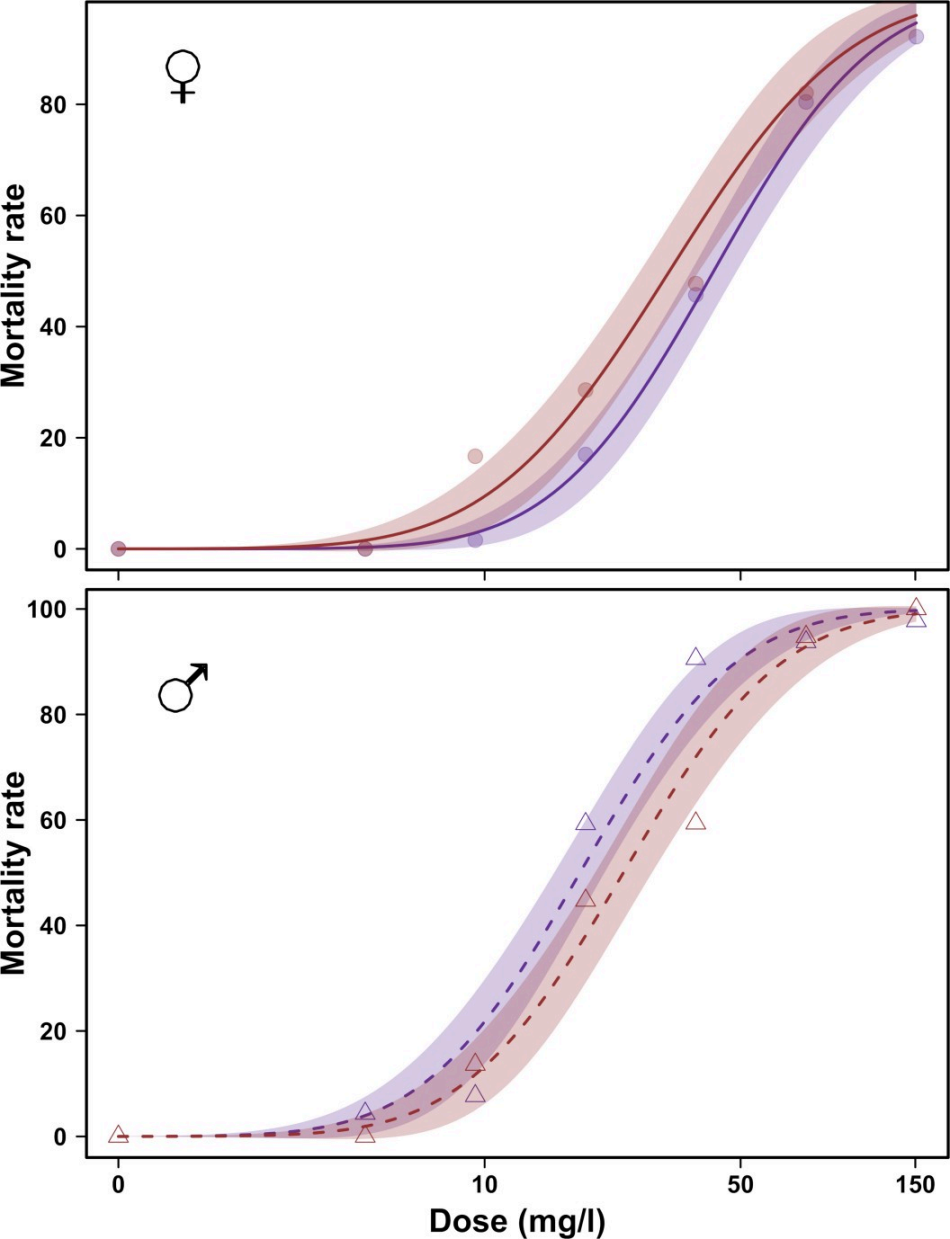
Effect of population genetic diversity on LD_50_ in *D*. *suzukii*. Dose-response curves of *D*. *suzukii* 24 to 48 hour-old adults of the population Ste-Foy (blue) and the inbred SF-IsoA (red) derived from it, after 24 h of tarsal exposure to phosmet in females (top panel) and males (bottom panel). The 95% confidence intervals were derived from the dose-response model.

The LD_50_ values estimated by dose-response curve analyses were very similar with overlapping 95% confidence intervals for females (31.9 mg/l (95% CI: 15.2–48.6) and 41.8 mg/l (95% CI: 35.7–48.0) for SF-IsoA and Ste-Foy, respectively) and males (22.4 mg/l (95% CI: 21.3–23.6) and 17.7 mg/l (95% CI: 13.2–22.3) for SF-IsoA and Ste-Foy, respectively) (Fig 4). Intra-population diversity did not influence the variations in our bioassays (relative potency for females SF-IsoA:Ste-Foy = 0.76, t-value = -1.12, p-value = 0.262 and relative potency for males SF-IsoA:Ste-Foy = 1.26, t-value = 1.57, p-value = 0.116).

### Effect of the experimental setup on LD_50_ values: Number of tested individuals and exposure duration

#### The influence of the number of tested individuals on insecticide susceptibility

**Fig 5:**
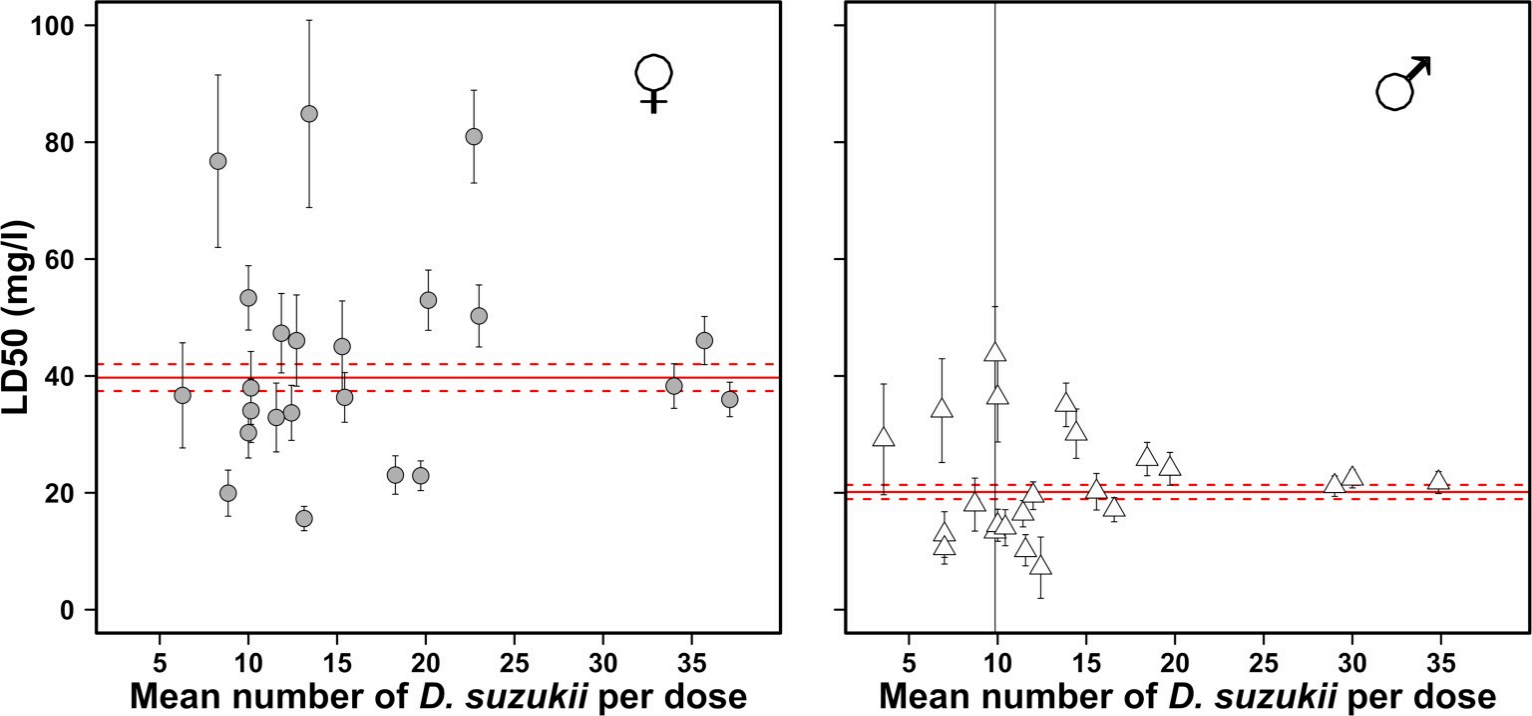
Effect of the mean number of individuals tested per dose on LD_50_ in a *D*. *suzukii* population. Distribution of LD_50_ values and associated 95% confidence intervals after 24 h of tarsal exposure of 24 to 48 hour-old adults to phosmet, according to the number of individuals tested for females (left panel) and males (right panel). The continuous red line indicates the LD_50_ value computed on pooled individuals as one unique bioassay and the dashed lines show the corresponding 95% confidence interval.

We analyzed the variation in the estimated LD_50_ values according to the number of tested individuals per bioassay per dose of phosmet (from a minimum mean number per dose of 9.4 and 10 flies for males and females, respectively, to a maximum mean number per dose of 34.9 and 37.1 for males and females, respectively). Our results clearly highlight the importance of the number of tested flies on the accuracy of the estimation of LD_50_ (Fig 5). For both females and males, an insufficient number of tested individuals led to imprecise and haphazard evaluations of LD_50_ (Fig 5). The standard errors associated with the LD_50_ values estimated with a limited number of individuals tended to be higher when fewer individuals were included in the experiment. In contrast, when the mean number of individuals tested per dose exceeded a threshold of approximatively 30, the LD_50_ assessed for the various bioassays were similar and close to the value estimated with every tested individual pooled together as one unique total bioassay according to sex. Males with 29, 30 and 35 individuals per dose led to a similar estimation of LD_50_ values with overlapping 95% confidence intervals: 21.1 mg/l (95% CI: 17.7–24.6), 22.4 mg/l (95% CI: 19.3–25.5) and 21.8 mg/l (95% CI: 18.1–25.4), respectively. Similarly, females gave LD_50_ estimates of 38.3 mg/l (95% CI: 30.8–45.8), 46.1 mg/l (95% CI: 38.0–54.1) and 36.0 mg/l (95% CI: 30.2–41.8), for respectively 34, 36 and 37 individuals per dose.

#### The influence of the duration of insecticide exposure on *D*. *suzukii* susceptibility

**Fig 6:**
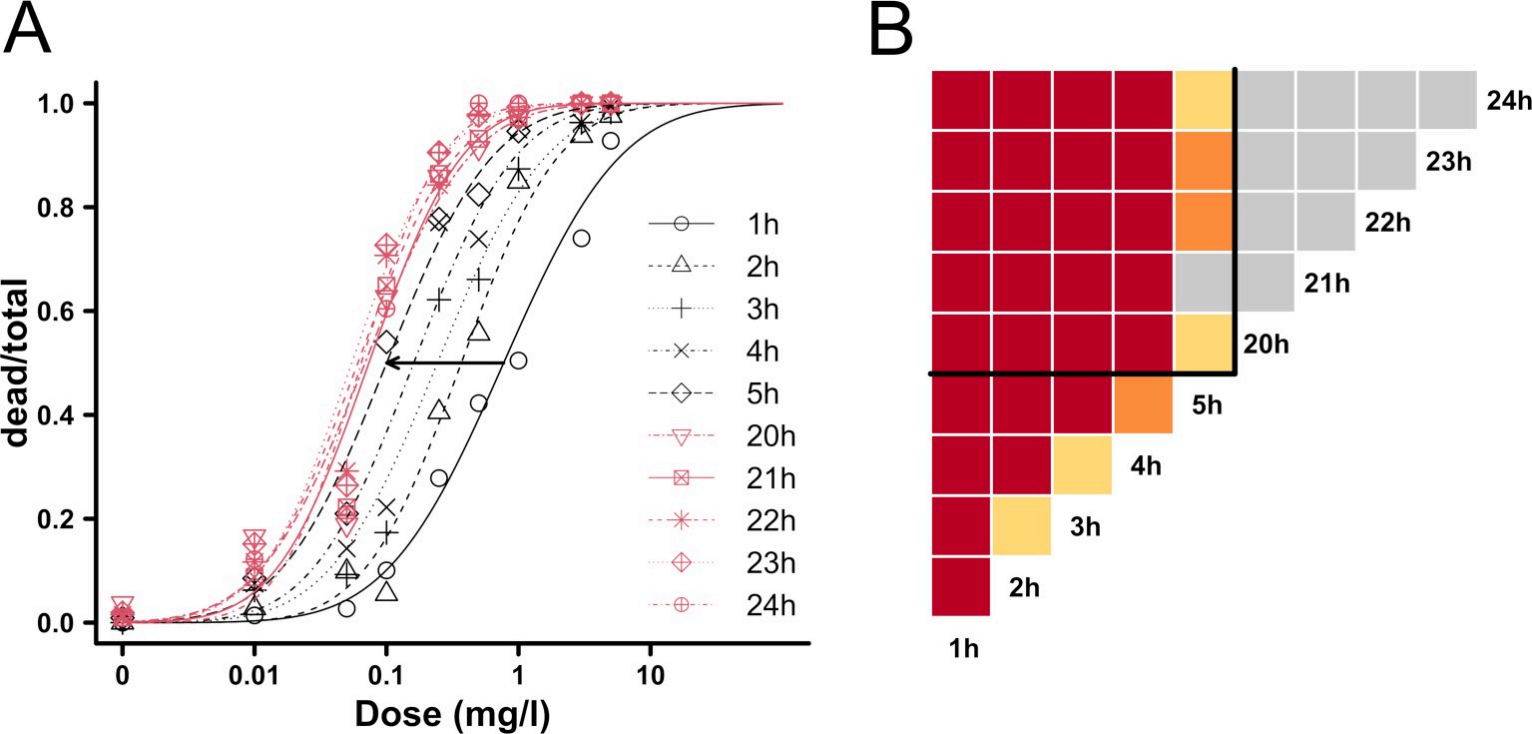
Effect of duration of exposure on LD_50_ estimates in a *D*. *suzukii* population. The bioassay consisted of a tarsal exposure to lambda-cyhalothrin and was conducted on 24 to 48 hour-old *D*. *suzukii* adults from the Ste-Foy population. Panel A shows the change in the dose-response curves for female individuals. Early scoring (after 1, 2, 3, 4 and 5 h) curves are shown in black and late scoring (after 20, 21, 22, 23 and 24 h) curves are shown in red. The black arrow shows the variation in LD_50_ between 1 and 5 h. The half-matrix in Panel B shows the significance levels of relative potencies between different exposure times for the female individuals (red: *p< 0*.*001*; orange: *0*.*001 ≤p <0*.*01*; yellow: *0*.*01 ≤ p <0*.*05*; gray: *ns*).

Exposure duration to lambda-cyhalothrin had a significant effect on the LD_50_ estimates in the study population, both for females and males. The three bioassays were very similar: only 2 out of 30 LD50 comparisons between bioassays at each time of exposure were significantly different. For this reason, subsequent analyses were performed on pooled data from the bioassays. During the first phase of the experiment (from 1 to 5 h duration), we observed a decrease in the LD_50_ estimate. In the second phase, the LD_50_ estimate was stable (from 20 to 24 h duration) (Fig 6A). The duration of exposure before scoring had a significant effect on the LD_50_ estimate (*e*.*g*. relative potency 5h:24h = 1.55, t-value = 2.53, p-value = 0.011) (Fig 6B). There was a clear significant break between 5 h and 20 h of exposure time. In particular, the estimation of LD_50_ for 1, 2, 3 or 4 h exposure was less precise than after longer exposure times, with larger standard errors and 95% confidence intervals. The LD_50_ estimates for 20 to 24 h of exposure were similar (relative potency close to 1) and not significantly different (Fig 6B).

#### Detection of putative mutations in the voltage-gated sodium channel

The genotyping of 20 random individuals from Ste-Foy and 20 individuals from Ste-Foy that survived for 24 h at 0.25 mg/l of lambda-cyhalothrin did not reveal the presence of mutant L1014F *kdr* alleles. Therefore, the results observed in our lambda-cyhalothrin assays were not biased by this mutation. The partial sequencing of the gene encoding for the voltage-gated sodium channel of two flies from the Ste-Foy population that survived at 0.25 mg/l of lambda-cyhalothrin and exhibited a *kdr*-like phenotype did not reveal the presence of any of the 12 main resistance mutations described in the literature (GenBank accession nos. MK645039-MK645040).

## Discussion

Our study explored a selection of critical parameters to control in pest bioassays. From an applied point of view, this study defined a useful reproducible protocol for a reliable tarsal-contact bioassay to evaluate insecticide resistance in *D*. *suzukii*.

We found that *D*. *suzukii* does not respond in the same way to insecticide according to sex and age. In most studies on insecticide resistance of *D*. *suzukii*, tests are done on females only or on a mix of males and females without taking sex into account. A few experiments have reported that males have greater insecticide susceptibility than females [44, 48, 52]. This phenomenon has also been observed in various insect species and specifically in Diptera for various insecticides, including pyrethroids and organophosphates [72–75]. We observed the same phenomenon in our experiment with males being twice as susceptible as females. The origin of this difference has already been discussed in other studies, being sometimes attributed to the difference in size between sexes due to sexual dimorphism [76–78], although detoxification enzymes also seem to be involved in these differences [79, 80]. Our results also showed an influence of age on susceptibility, but only for females. Susceptibility to pesticide can vary according to the development stage of the insect [81]. Smirle *et al*. [48] reported that malathion (an organophosphate) induces more toxicity on 5–8-day-old *D*. *suzukii* adults than on 2-day-old individuals. Most experiments use flies at least 5 days old, which is time-consuming in terms of sample preparation. No experiment has indicated insecticide susceptibility in *D*. *suzukii* flies younger than 24 h. In our experiment, newly hatched females (0–24 h) showed significantly higher susceptibility to insecticides than older age classes. This period corresponds to the sexual maturation stage in this synovigenic species [82, 83]. During the ovarian maturation period, metabolic detoxification pathways and energy investments are different compared with the rest of the adult life [84, 85]. Testing the effects of mating status on insecticide resistance might be an interesting avenue of research. Mating occurs mainly after the female refractory period of 1 to 2 days after emergence [82, 86] and generates strong changes in female physiology and behavior in *Drosophila* [87, 88]. On the contrary, young *D*. *melanogaster* flies have been described as more resistant to several abiotic stresses [89]. Our finding is of interest for establishing a reliable bioassay method, but may also have an applied interest in targeting treatments on the early life stage of young females.

Inbred populations with low genetic diversity may display lower resistance to pathogenic organisms [90] and may be more sensitive to environmental stress [91]. By creating a low genetic diversity population SF-IsoA, we were able to test the hypothesis that reduced genetic diversity may (i) reduce the variability of insecticide response estimates (i.e. LD_50_ values) within the population and increase repeatability, and (ii) genetically fix the strain and obtain a stable reference population through generations. We therefore expected a different insecticide response in the SF-IsoA compared with the Ste-Foy population. However, no differences in susceptibility were detected in our comparison between original and inbred populations. Our finding is thus likely an illustration of the fact that the Ste-Foy population is initially devoid of resistance alleles and therefore allelic paucity does not change this status.

Regarding the effect of the experimental setup on insecticide susceptibility, the number of tested flies per bioassay and per dose and the duration of exposure to the tested insecticide are essential parameters to control. As suggested in previous studies, a range from 10 to 25 insects per dose is necessary [92, 93]. Our results showed that the number of tested insects is the most important factor among those explored in this work. According to our data, a reliable mean number of tested insects per dose is a minimum of 29 males and 34 females. We therefore recommend about 30 individuals per dose for the tests to be accurate and reproducible. For most of the existing protocols on *D*. *suzukii*, the duration of exposure to the chemical is usually about 24 h [44, 48, 50, 51] and can reach 48 or 72 h [4, 43, 45, 47]. A study on the mortality induced by ingestion of spinosad on this species emphasized the importance of time elapsed (5 days) before assessing mortality, suggesting that exposure duration is crucial in different types of bioassays [53]. Bioassays can be extremely time consuming, making it tempting to minimize exposure duration to reduce the total testing time. However, for pyrethroids, several species have shown a knockdown phenotype that can induce a transient state of apparent mortality in the insects a few minutes after exposure to the insecticide. In a study of mosquitoes exposed to lambda-cyhalothrin, the time to 50% knockdown (KT_50_) ranged from 14 (*Mansonia africana*) to 128 min (*Anopheles gambiae*) with KT_95_ values of 42 and 277 min, respectively [94]. In another study, the KT_50_ for *D*. *melanogaster* flies ranged from 11 to 40 min, depending on the pyrethroid and the strain [95]. Therefore, assessing the mortality too early after exposure may distort results, while a late, albeit reliable, assessment [96] may result in lost time. We observed a few individuals with a *kdr*-like phenotype in our experiment. They showed temporary paralysis followed by complete recovery of their locomotive capacities. Additional research did not reveal any of the main resistance mutations or the *kdr* mutation at the expected locus (1014) of the *para* gene. Similar results have been reported from a study on *D*. *suzukii* adults exposed to spinosad [52]–to which resistance is emerging in the US [37]. This study’s results showed highly variable LD_50_ values, calculated from the large number of moribund flies due to an insufficient exposure time (6 h). To avoid potential misinterpretation due to a knockdown effect, a rapid detoxification mechanism (potentially present in susceptible insects) or a slow mode of action of an active substance, we recommend assessing insect mortality at least 24 h after insecticide exposure (or more depending on the mode of action of the insecticide). Because the phenotypic effect of the insecticide is estimated by visual assessment, it is of utmost importance to establish precise scoring criteria that can be standardized across experiments and laboratory workers, in particular to differentiate moribund individuals from the dead and live individuals.

As illustrated by the low to moderate levels of resistance of *D*. *suzukii* populations that have been described in the US [37], monitoring insecticide resistance of this pest is important to be able to react quickly and adapt control management. We hereby propose a validated and operational method for a reliable worldwide monitoring of *D*. *suzukii* resistance to insecticides, and highlighted several parameters that are essential to control for in the design of resistance assessment bioassays on other species as well.

## Supporting information

S1 Table Thirteen microsatellites markers used in *Experiment 3*: Observing the impact of the genetic diversity of a population on its resistance to phosmet. (DOCX)

S2 Table Diversity index for two *Drosophila suzukii* populations: Ste-Foy and SF-IsoA (*Experiment 3*). (DOCX)

S1 Fig Schematic representation of the protein subunit and gene exon-intron organization for the trans-membrane segments of the voltage-gated sodium channel (domain II, α subunit) showing the most frequent non-synonymous mutations as well as the PCR and sequencing primer positions (*Experiment 5*). (DOCX)
