# Supplementary material for: How varying parameters impact insecticide resistance bioassay: An example on the worldwide invasive pest Drosophila suzukii
Source: PLoS One. 2021 Mar 5;16(3):e0247756. doi: 10.1371/journal.pone.0247756 (PMC7935283; doi:10.1371/journal.pone.0247756)
Supplement: S1 Table — (DOCX) [file pone.0247756.s001.docx]

**S1 Table.** **Thirteen** **microsatellites markers used in *Experiment 3*: observing the impact of the genetic diversity of a population on its resistance to phosmet.**

| **Locus** | **Repeat** | **Primer sequence 5'→3'** | **Dye** | **Size range (bp)** |
| --- | --- | --- | --- | --- |
| DS_05 | (TG)10 | F: AGGATAACGCGCAGCTTGAC | Atto565 | 260–300 |
|  |  | R: TATGGAAGCTGGCAAGCAGA |  |  |
| DS_07 | (CA)13 | F: AAGGCTGGAGTGGCAACAA | Atto565 | 160–210 |
|  |  | R: GCTAAGGTTCTGTTCGGCTG |  |  |
| DS_08 | (AG)10 | F: CGTTGTTGGCGGTGAGTAAG | Tamra | 110–170 |
|  |  | R: GGCCATCAATCAGTCAGTCA |  |  |
| DS_09 | (AC)15 | F: CACACATGGCGTATGCGTAT | Fam | 190–250 |
|  |  | R: ACTTGTTGAGCCGTCCTGG |  |  |
| DS_12 | (GT)19 | F: GCTGTTGCTGTTGCTATTGC | Atto565 | 320–380 |
|  |  | R: AGAACCGTTAGCTGAGCGAG |  |  |
| DS_14 | (TG)10 | F: AAGAACCGCAACGAGCAA | Tamra | 180–220 |
|  |  | R: GAATTATCCAGCGACACGAC |  |  |
| DS_15 | (GT)11 | F: GGACAGCCGACATAAGAGG | Fam | 260–320 |
|  |  | R: GAGTTGCTGGCTCGACACTT |  |  |
| DS_16 | (AC)13 | F: TTCGTATGTTAGGCGCCA | Fam | 100–140 |
|  |  | R: CTGGCTGCTGACCTCAACTC |  |  |
| DS_17 | (GT)10 | F: CATCTCAGGCCACGAATG | Atto565 | 80–130 |
|  |  | R: CTCCAGATTCTCGAGTGCAG |  |  |
| DS_20 | (AG)12 | F: CAGCCATATGCAATGCACTG | Hex | 210–270 |
|  |  | R: ATATCCAGCGGAAGTCGAGA |  |  |
| DS_27 | (GT)14 | F: CCAGCGACTGCAGAAGTGAC | Hex | 80–130 |
|  |  | R: GCAATCCTCCACAACACAAC |  |  |
| DS_28 | (TG)11 | F: TTAAGCTGACCTCCTCCTCG | Hex | 140–195 |
|  |  | R: GCACTCGCACAGATACAAGG |  |  |
| DS_32 | (TG)15 | F: CGGCGTGTTGCAGTTATTC | Fam | 330–380 |
|  |  | R: ATGCACTGGTCGACATGACA |  |  |

Locus – marker name (Fraimout *et al.* 2015), Repeat – microsatellite motif, Primer sequence – F: forward and R: reverse, Dye – fluorescent dye used to label universal M13-tail primer, 6-FAM (6-carboxyfluorescein), HEX (hexachloro-fluoresceine), Tamra (carboxy-tetramethyl-rhodamine), ATTO 565 (rhodamine dye), Size Range – allele size range in base pairs (bp).

**Reference**

Fraimout A, Loiseau A, Price DK, Xuéreb A, Martin J-F, Vitalis R, et al. New set of microsatellite markers for the spotted-wing *Drosophila suzukii* (Diptera: Drosophilidae): a promising molecular tool for inferring the invasion history of this major insect pest. European Journal of Entomology. 2015;112(4):855-59.
