# Supplementary material for: How varying parameters impact insecticide resistance bioassay: An example on the worldwide invasive pest Drosophila suzukii
Source: PLoS One. 2021 Mar 5;16(3):e0247756. doi: 10.1371/journal.pone.0247756 (PMC7935283; doi:10.1371/journal.pone.0247756)
Supplement: S2 Table — (DOCX) [file pone.0247756.s002.docx]

**S2 Table. Diversity index for two *Drosophila suzukii* populations*:* Ste-Foy and SF-IsoA (*Experiment 3*).**

|  | **SF-IsoA**  **(N=44; 30♀, 14♂)** | | | **Ste_Foy**  **(N=44; 30♀, 14♂)** | | |
| --- | --- | --- | --- | --- | --- | --- |
| **Locus** | ***N*_a_** | ***H*_e_** | ***H*_o_** | ***N*_a_** | ***H*_e_** | ***H*_o_** |
| **Ds_05** | 1 | 0 | 0 | 4 | 0.605 | 0.585 |
| **Ds_07** | 2 | 0.464 | 0.500 | 5 | 0.731 | 0.780 |
| **Ds_08** | 3 | 0.673 | 0.737 | 3 | 0.446 | 0.439 |
| **Ds_09** | 2 | 0.462 | 0.486 | 4 | 0.653 | 0.634 |
| **Ds_12** | 1 | 0 | 0 | 3 | 0.642 | 0.410 |
| **Ds_14** | 1 | 0 | 0 | 3 | 0.645 | 0.512 |
| **Ds_15** | 1 | 0 | 0 | 3 | 0.594 | 0.375 |
| **Ds_16** | 1 | 0 | 0 | 2 | 0.447 | 0.317 |
| **Ds_17** | 1 | 0 | 0 | 3 | 0.585 | 0.512 |
| **Ds_20** | 2 | 0.321 | 0.237 | 2 | 0.429 | 0.366 |
| **Ds_27** | 2 | 0.405 | 0.395 | 4 | 0.695 | 0.585 |
| **Ds_28** | 1 | 0 | 0 | 3 | 0.583 | 0.550 |
| **Ds_32** | 1 | 0 | 0 | 4 | 0.683 | 0.585 |
| **Mean ±SD** | **1.462 ±0.66** | **0.179 ±0.247** | **0.181 ±0.261** | **3.308 ±0.855** | **0.595 ±0.098** | **0.512 ±0.129** |

*N*_a_ – Allele number, *H*_o_ – observed heterozygosity for each microsatellite locus; *H_e_* – expected heterozygosity, N – number of sampled individuals. Calculated using GENEPOP V4.2.2 (Rousset, 2008)

**Reference**

Rousset F. genepop’007: a complete re‐implementation of the genepop software for Windows and Linux. Molecular ecology resources. 2008;8(1):103-6.
