# Supplementary material for: How varying parameters impact insecticide resistance bioassay: An example on the worldwide invasive pest Drosophila suzukii
Source: PLoS One. 2021 Mar 5;16(3):e0247756. doi: 10.1371/journal.pone.0247756 (PMC7935283; doi:10.1371/journal.pone.0247756)

**S1 Fig. Schematic representation of the protein subunit and gene exon-intron organization for the trans-membrane segments of the voltage-gated sodium channel (domain II, α subunit) showing the most frequent non-synonymous mutations as well as the PCR and sequencing primer positions (*Experiment 5*).** Crosses indicate the positions of the major non-synonymous mutations: L1014F (*kdr*), M918T (Super *kdr*) and secondary mutations L925I, T929I, L932F, C933A and I936V. Arrows indicate the positions of the primers used for sequencing and PCR-RFLP analyses (see Table 1)


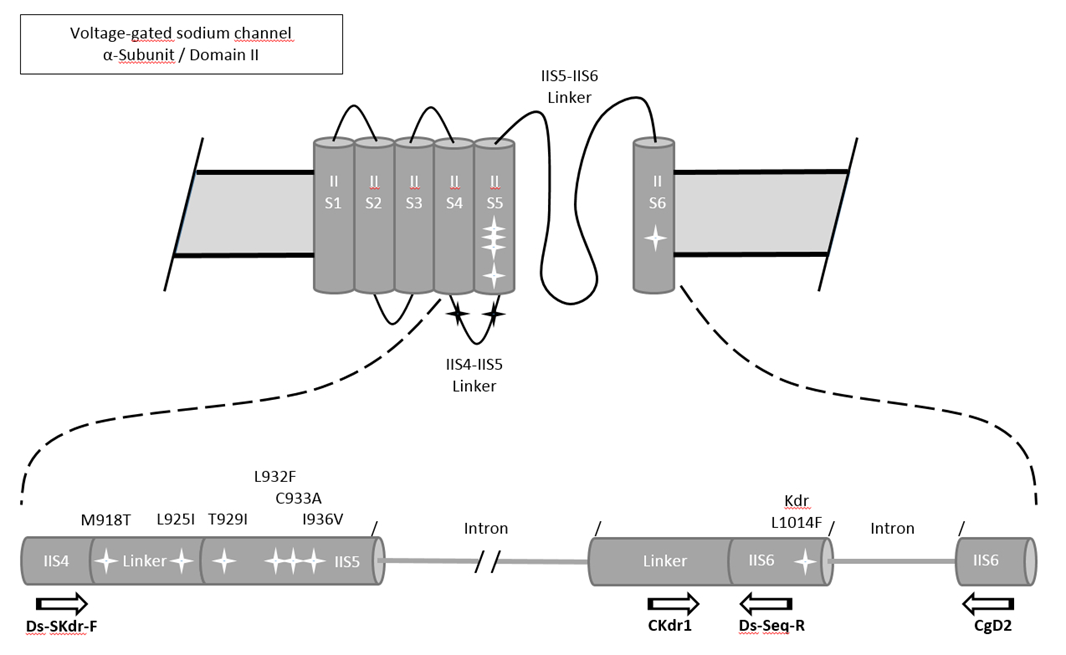

Supplement: S1 Fig — (DOCX) [file pone.0247756.s003.docx]
