# Supplementary material for: How varying parameters impact insecticide resistance bioassay: An example on the worldwide invasive pest Drosophila suzukii
Source: PLoS One. 2021 Mar 5;16(3):e0247756. doi: 10.1371/journal.pone.0247756 (PMC7935283; doi:10.1371/journal.pone.0247756)
Supplement: Peer review history

# Peer review: Decision Letter 0

**DOI:** 10.1371/journal.pone.0247756.r001

**Authors:** Maohua Chen

10 Jul 2020

PONE-D-20-13087

How to create a reliable and reproducible insecticide resistance bioassay: an example on the worldwide invasive pest Drosophila suzukii

PLOS ONE

Dear Dr. Benoit Barres,

Thank you for submitting your manuscript to PLOS ONE. After careful consideration, we feel that it has merit but does not fully meet PLOS ONE’s publication criteria as it currently stands. Therefore, we invite you to submit a revised version of the manuscript that addresses the points raised during the review process.

Please submit your revised manuscript by 10 August 2020. If you will need more time than this to complete your revisions, please reply to this message or contact the journal office at plosone@plos.org. When you're ready to submit your revision, log on to https://www.editorialmanager.com/pone/ and select the 'Submissions Needing Revision' folder to locate your manuscript file.

Please include the following items when submitting your revised manuscript:

- A rebuttal letter that responds to each point raised by the academic editor and reviewer(s). You should upload this letter as a separate file labeled 'Response to Reviewers'.
- A marked-up copy of your manuscript that highlights changes made to the original version. You should upload this as a separate file labeled 'Revised Manuscript with Track Changes'.
- An unmarked version of your revised paper without tracked changes. You should upload this as a separate file labeled 'Manuscript'.

If you would like to make changes to your financial disclosure, please include your updated statement in your cover letter. Guidelines for resubmitting your figure files are available below the reviewer comments at the end of this letter.

If applicable, we recommend that you deposit your laboratory protocols in protocols.io to enhance the reproducibility of your results. Protocols.io assigns your protocol its own identifier (DOI) so that it can be cited independently in the future. For instructions see: http://journals.plos.org/plosone/s/submission-guidelines#loc-laboratory-protocols

We look forward to receiving your revised manuscript.

Kind regards,

Maohua Chen, Ph.D

Academic Editor

PLOS ONE

Journal Requirements:

Journal requirements:

When submitting your revision, we need you to address these additional requirements.

Please ensure that your manuscript meets PLOS ONE's style requirements, including those for file naming. The PLOS ONE style templates can be found at

https://journals.plos.org/plosone/s/file?id=wjVg/PLOSOne_formatting_sample_main_body.pdf and

https://journals.plos.org/plosone/s/file?id=ba62/PLOSOne_formatting_sample_title_authors_affiliations.pdf

[Note: HTML markup is below. Please do not edit.]

Reviewers' comments:

Reviewer's Responses to Questions

**Comments to the Author**

1. Is the manuscript technically sound, and do the data support the conclusions?

The manuscript must describe a technically sound piece of scientific research with data that supports the conclusions. Experiments must have been conducted rigorously, with appropriate controls, replication, and sample sizes. The conclusions must be drawn appropriately based on the data presented. 

Reviewer #1: Partly

**********

2. Has the statistical analysis been performed appropriately and rigorously? 

Reviewer #1: Yes

**********

3. Have the authors made all data underlying the findings in their manuscript fully available?

The PLOS Data policy requires authors to make all data underlying the findings described in their manuscript fully available without restriction, with rare exception (please refer to the Data Availability Statement in the manuscript PDF file). The data should be provided as part of the manuscript or its supporting information, or deposited to a public repository. For example, in addition to summary statistics, the data points behind means, medians and variance measures should be available. If there are restrictions on publicly sharing data—e.g. participant privacy or use of data from a third party—those must be specified.

Reviewer #1: Yes

**********

4. Is the manuscript presented in an intelligible fashion and written in standard English?

PLOS ONE does not copyedit accepted manuscripts, so the language in submitted articles must be clear, correct, and unambiguous. Any typographical or grammatical errors should be corrected at revision, so please note any specific errors here.

Reviewer #1: Yes

**********

5. Review Comments to the Author

Please use the space provided to explain your answers to the questions above. You may also include additional comments for the author, including concerns about dual publication, research ethics, or publication ethics. (Please upload your review as an attachment if it exceeds 20,000 characters)

Reviewer #1: I was interested in the topic of this manuscript as I do believe there are issues with several methods being used to monitor resistance in SWD. After reading the manuscript I don’t think the title is fitting. I wonder if something more along the lines of ‘How varying parameters impact insecticide resistance monitoring: an example… ‘How to create’ implies that this should be easily reproducible methodology and instructions on a step by step process, but the lack of detail in the methodology leaves room for variation. What the manuscript does is highlight the variation in insecticide tolerance bioassays results based on some parameters. It does not document the optimum bioassay- which the current title implies.

Another key point is I think the term replicate is misleading in this manuscript. For me a replicate is a repeat of a particular set of parameters in which there is no difference between them. For example in experiment 4 the whole point of the experiment is to have different numbers of flies to explore how this impacts the LC50 value. However, the numbers appear to be completely random even between the ‘replicates’ indicated by giving the mean in the S1 table and the range of numbers in each vial isn’t very diverse. Why was there no systematic choice of number of individuals? Also why not even numbers of males and females within ‘replicates’? It just gives the impression there has been very little thought to statistical analysis and the actual question you suggest you are asking. It seems like you did experiments and then though what can we look at with this data.

Why was only one experiment carried out for lambda cyhalothrin? It’s a shame experiments 1-4 did not use lambda cyhalothrin as it would have been interesting to see if males and females and different ages are as variable in their responses as they are to phosmet. I understand the comment about knock down effect but the fact that there is only one rep for this experiment it seems like a last minute add on. I’d seriously consider removing the lambda cyhalothrin experiments from this manuscript and saving it when the work can be repeated and can be and explored fully.

Overall I found the manuscript generally easy to follow and the language accessible and clear, but had to keep going back to the methodology. I kept thinking I had missed information but it was just that it wasn’t included or clear in the text. Frequently there is no indication of numbers of males and females, how old flies were for each experiment and it is only from parts of the results that some of these points are address. I have more specific points below.

L110- why 3 generations of inbreeding. Do you have a reference that shows this is enough for an iso line?

L119- dimensions of the vials?

L128- at first I thought you were stating ranges but these are specific doses. Can you change the ‘–‘ to ‘,’ to show it is a list.

L132- from what amount of flies? Could be 1-45 or 30-45, although this is included in S1 its helpful to give an indication to the reader. Also, although you state age later in the methods can you include the standard age used.

L134- How were flies added to the vials if they were not anesthetized? Sex can be identified without having to knock out the flies. You state this yourself when you do the mortality assessments, on the presence of the wing spot. Even if you are working with flies less than 24 hours old you can still sex them visually by the presence of the sex combs which are present at eclosion. The female ovipositor is also obvious.

L136- what environmental conditions were they subjected to during the exposure to the insecticides?

L144-159- what do you mean by replicates that are stated in the brackets? Are these the total number of vials tested or the replicates of each treatment? If it is the former then this is very miss leading. Add the treatments and number of true replicates stated in additional supplementary figures as you have for experiment 4.

L-149- what age of flies were used for the experiment?

L151- how did you divide males and females?

L155- what insecticide is used for experiment 4?

L158- 1 replicate… not scientifically sound. Did you start off with more reps but due to high control mortality have to remove them from the analysis? If so then state this. How many males and females in each rep. Why 25 flies when you found in experiment 1 that above 30 flies gave a good indication of population susceptibility?

L162- did you only observe one vial throughout the whole experiment or are there 10 vials that are looked at once at defined times? If so shaking the vial every hour to check mortality couldn’t have been good for fly health.

L168- what ages were the flies used for extractions? Were they killed prior to being extracted and if so how? How long had they been dead before they were used and were they stored in anything? Storage and treatment can impact the DNA quality.

L261- how can you do stats on 1 rep? I am a reader would not rely on the results from 1 repetition.

L274- add ‘phosmet’ in the second sentence to read ‘The LD50 values for phosmet were…’

L275- explain what the figures in the brackets are.

L284- in this experiment you have in effect several parameters that are not constant. You only discuss age of flies but what about their mating status? The youngest age group will not have mated but the other two are likely to have. I know in the discussion you talk about sexual maturity but what about the differences of mating status on susceptibility. Mated and unmated females have different behavioral patterns Ferguson, Calum TJ, et al. "The sexually dimorphic behaviour of adult Drosophila suzukii: elevated female locomotor activity and loss of siesta is a post-mating response." Journal of experimental biology 218.23 (2015): 3855-3861. Is there any evidence that mating status impacts tolerance?

L285- Remove ‘mild but’ from sentence.

L334- what active are you talking about?

L345- again replicates… it would be better to have table S1 in the actual manuscript but make it clearer how many repetitions of each number of flies you have. Do you have 5 replicates of 29 males? It’s just not very clear as it makes me suspicious that there is actually only one replicate of 29.

L395- You don’t explain in the introduction, method or results that pyrethroids target the sodium channel - this as a key point. Why didn’t you do the experiments with phosmet? You need to make it clearer why you did what you did. Readers who are not aware of MOA’s will not understand why you did this work on this active.

L463- Assessing mortality too early may distort results- also mortality does not always result in population reduction. Look at the paper Shaw, B., et al. (2019). "Implications of sub-lethal rates of insecticides and daily time of application on Drosophila suzukii lifecycle." Crop Protection 121: 182-194. Several elements are relevant to your manuscript.

**********

6. PLOS authors have the option to publish the peer review history of their article (what does this mean?). If published, this will include your full peer review and any attached files.

If you choose “no”, your identity will remain anonymous but your review may still be made public.

**Do you want your identity to be public for this peer review?** For information about this choice, including consent withdrawal, please see our Privacy Policy.

Reviewer #1: No

[NOTE: If reviewer comments were submitted as an attachment file, they will be attached to this email and accessible via the submission site. Please log into your account, locate the manuscript record, and check for the action link "View Attachments". If this link does not appear, there are no attachment files.]

While revising your submission, please upload your figure files to the Preflight Analysis and Conversion Engine (PACE) digital diagnostic tool, https://pacev2.apexcovantage.com/. PACE helps ensure that figures meet PLOS requirements. To use PACE, you must first register as a user. Registration is free. Then, login and navigate to the UPLOAD tab, where you will find detailed instructions on how to use the tool. If you encounter any issues or have any questions when using PACE, please email PLOS at figures@plos.org. Please note that Supporting Information files do not need this step.

# Author comment: Author response to Decision Letter 0

**DOI:** 10.1371/journal.pone.0247756.r002

29 Dec 2020

Response to reviewer’s comments to author

Reviewer #1: 

General comments

Response: we thank the reviewer for the careful and thoughtful review of our manuscript. The reviewer raised some important comments/questions. We have edited the manuscript following comments from this reviewer and hope that the revisions help to clarify points of confusion and improve the manuscript’s quality. 

I was interested in the topic of this manuscript as I do believe there are issues with several methods being used to monitor resistance in SWD. After reading the manuscript I don’t think the title is fitting. I wonder if something more along the lines of ‘How varying parameters impact insecticide resistance monitoring: an example… ‘How to create’ implies that this should be easily reproducible methodology and instructions on a step by step process, but the lack of detail in the methodology leaves room for variation. What the manuscript does is highlight the variation in insecticide tolerance bioassays results based on some parameters. It does not document the optimum bioassay- which the current title implies. 

Response: we agree with the reviewer comment. In order to have a title that better reflects the content of the manuscript, we changed it to: “How varying parameters impact insecticide resistance bioassay: an example on the worldwide invasive pest Drosophila suzukii”. 

Another key point is I think the term replicate is misleading in this manuscript. For me a replicate is a repeat of a particular set of parameters in which there is no difference between them. For example in experiment 4 the whole point of the experiment is to have different numbers of flies to explore how this impacts the LC50 value. However, the numbers appear to be completely random even between the ‘replicates’ indicated by giving the mean in the S1 table and the range of numbers in each vial isn’t very diverse. Why was there no systematic choice of number of individuals? 

Response: we agree with the reviewer that the term ‘replicate’ was misleading. The term was replaced by ‘bioassay’. Bioassays are not true experimental replications but are tests that are conducted on the same date using one or several vials per dose. The parameters used for the different bioassays can vary. The number of flies per vial was not controlled precisely for two reasons: (i) in order to limite the risk to damage flies through anesthesia or by manipulating them, the flies were transferred directly from the rearing vials to the test vials by connecting them with a small funnel. As the flies were awake and dynamic, control of the number of transferred flies per vial was limited (ii) the production of individuals in rearing vials was not constant over time, which sometimes constrained the number of individuals tested. The sexing and accurate counting of flies was carried out at the same time as the assessment of mortality. This protocol explains why the number of flies per vial was variable and can appear somewhat ‘random’. We tried to make this explanation clearer in the manuscript. We think this is not a major issue for the Experiment 4 since the aim of this experiment was to assess the effect of the number of individuals tested on the precision of the LD50 estimate: precise control of the mean number of individuals per dose was not necessary for this assessment, as long as variation existed. 

Also why not even numbers of males and females within ‘replicates’? It just gives the impression there has been very little thought to statistical analysis and the actual question you suggest you are asking. It seems like you did experiments and then though what can we look at with this data. 

Response: there was not an even numbers of males and females within bioassays because of our sorting and sexing protocol. As mentioned previously, we wanted to avoid a sexing before the bioassay because it implies an anesthesia with CO2 or nitrogen, or the use of ice to send the flies to sleep. We feared that it would impair the flies. Another way of sorting the flies would be to use a mouth aspirator, but again there was a risk of an impact on the flies. We rather didn’t separate males and females before the experiment. 

Why was only one experiment carried out for lambda cyhalothrin? It’s a shame experiments 1-4 did not use lambda cyhalothrin as it would have been interesting to see if males and females and different ages are as variable in their responses as they are to phosmet. I understand the comment about knock down effect but the fact that there is only one rep for this experiment it seems like a last minute add on. I’d seriously consider removing the lambda cyhalothrin experiments from this manuscript and saving it when the work can be repeated and can be and explored fully.

Response: we thank the reviewer for pointing out this weakness in the design of the experiment, especially concerning the lack of repetition for Experiment 4. Concerning the experiment on the duration of insecticide exposure, we knew that pyrethroids can induce mortality from few minutes or hours and even a knock down in some cases. We thought it would be more interesting to test the duration of exposure with a pyrethroid (lambda-cyhalothrin) than an organophosphate (phosmet). We agree that only one repetition for this experiment was not consistent enough, that is why we decided to repeat it (3 times) after reading your review. These new bioassays gave consistent results with one another. For both the new and original bioassays the LD50 estimates were found to decrease during early stages (between 1 to 5 h) of the experiment and then stabilized after 20 to 24 hours. Contrary to what we recorded in the original bioassay, we did not observe an increase of the LD50 between the end of the first and the beginning of the second time span of the experiment. Also, the LD50 of the Ste-Foy population is sligthly lower this year than in 2017. It might be due to the evolution of Ste-Foy population during the last 3 years. Because the results were more consistent for the new bioassays and because we could not reproduce the exact same results of the experiment of 2017, we decided to replace this latter bioassay by the 3 repetitions performed this year in the manuscript. This does not affect the general conclusion drawn from this experiment. 

Overall I found the manuscript generally easy to follow and the language accessible and clear, but had to keep going back to the methodology. I kept thinking I had missed information but it was just that it wasn’t included or clear in the text. Frequently there is no indication of numbers of males and females, how old flies were for each experiment and it is only from parts of the results that some of these points are address. I have more specific points below. 

Response: thank you for this warning, we added more details about the number of flies, their age and other parameters for each experiment in a clearer manner in ‘Materials and Methods’ (see response to specific points below). A new table has also been added to this section to summarize all the conditions of the different experiments (Table 1). 

Specific points: 

L110- why 3 generations of inbreeding. Do you have a reference that shows this is enough for an iso line? 

Response: this is a good remark and we have reconsidered the use of the term iso line since it doesn’t fit the usual definition used by groups working on Drosophila. Three generatios of inbreeding between full sister and brother lead to an inbreeding coefficient of nearly 60% (this is conservative estimate considering that the inbreeding coefficient of the population from which the first brother and sister couple were drown from was 0). But the reviewer is right, usually a line is considered as isogenic after 10 to 15 inbreeding crosses. Our aim here was to test the effect of a drastic depletion of the original genetic diversity on LD50 estimation. Considering the genetic diversity level before and after the 3 generations of inbreedings as measured with microsatellites, we believe that our approach is still relevant. We therefore replaced “A female inbred line” (line 109 of the original submission) and “isoline” (used line 436 of the original submission) by “A low genetic diversity population”. 

L119- dimensions of the vials? 

Response: the dimensions of the testing vials were ø x h : 28 x 61 mm with the cap for a volume of 20 ml and we added it (line 119 of the unmarked revised version). 

L128- at first I thought you were stating ranges but these are specific doses. Can you change the ‘–‘ to ‘,’ to show it is a list. 

Response: done. 

L132- from what amount of flies? Could be 1-45 or 30-45, although this is included in S1 its helpful to give an indication to the reader. Also, although you state age later in the methods can you include the standard age used. 

Response: the range of the number of flies has been added as well as the age (lines 135-136 of the unmarked revised version). 

L134- How were flies added to the vials if they were not anesthetized? Sex can be identified without having to knock out the flies. You state this yourself when you do the mortality assessments, on the presence of the wing spot. Even if you are working with flies less than 24 hours old you can still sex them visually by the presence of the sex combs which are present at eclosion. The female ovipositor is also obvious. 

Response: the flies were transferred from the rearing vials to the test vials using a small funnel, pressing the edges of the vial onto the plastic funnel to prevent the flies from escaping. Although sexing would have been possible without stunning the flies, it would have been difficult to sort them into separate vials while they were still awake without risking injury. That is why we did not sort them before the bioassay. We just transferred individuals into the testing vial and we did the sexing at the same time as the mortality assessment. This method has the advantages of (i) saving time (ii) limiting the risk to damage flies by the anesthesia or by manipulating them. The sexing after 24h of exposition to the insecticide is also a lot easier, thanks to the black dots on the male wings. The protocole is easier to transfer to other laboratories even if they lack expertise in D. suzukii (the observation of the sex combs needs more practice). 

L136- what environmental conditions were they subjected to during the exposure to the insecticides? 

Response: this is an oversight, thank you for noticing. We added the rearing temperature and the duration of the light-dark cycle (line 140 of the unmarked revised version). 

L144-159- what do you mean by replicates that are stated in the brackets? Are these the total number of vials tested or the replicates of each treatment? If it is the former then this is very miss leading. Add the treatments and number of true replicates stated in additional supplementary figures as you have for experiment 4.

Response: the term ‘replicate’ have been replaced by ‘bioassay’ throughout the manuscript. The Table 2 (former S1 Table) summarize all the bioassays performed for each experiment as advised by the reviewer. 

L-149- what age of flies were used for the experiment? 

Response: the age of the flies was 24 to 48h, it has been added in the text. 

L151- how did you divide males and females? 

Response: this is an error of wording. We meant that the flies (males and females) were of three different age classes. This has been reformulated in the manuscript. 

L155- what insecticide is used for experiment 4? 

Response: the insecticide was phosmet, the information has been added (line 165 of the unmarked revised version). 

L158- 1 replicate… not scientifically sound. Did you start off with more reps but due to high control mortality have to remove them from the analysis? If so then state this. How many males and females in each rep. Why 25 flies when you found in experiment 1 that above 30 flies gave a good indication of population susceptibility? 

Response: see the response in the general comment section. Three new true repetitions were made and added to the manuscript. 

L162- did you only observe one vial throughout the whole experiment or are there 10 vials that are looked at once at defined times? If so shaking the vial every hour to check mortality couldn’t have been good for fly health. 

Response: the same vials were looked at each defined times. We decided it was preferable to observe the evolution of mortality according to time on the same individuals. One small tap with the finger was made on the vial each time mortality was recorded (10 times) but we do not think it has a significant impact on fly health. Indeed, the flies in the control vials were all alive and stayed in good health with an increased time of exposition to acetone alone (control). We added “..was assessed repeatedly on the same vials at 10 different times…” (lines 171-172 of the unmarked revised version). 

L168- what ages were the flies used for extractions? Were they killed prior to being extracted and if so how? How long had they been dead before they were used and were they stored in anything? Storage and treatment can impact the DNA quality

Response: thank you for pointing out that details were missing. Indeed storage and treatment can impact DNA quality, we added more specifics to the text (lines 183-185 and lines 221-224). 

L261- how can you do stats on 1 rep? I am a reader would not rely on the results from 1 repetition.

Response: this comment is not relevant anymore since the single replicate was replaced by three new replicates. 

L274- add ‘phosmet’ in the second sentence to read ‘The LD50 values for phosmet were…’

Response: done. 

L275- explain what the figures in the brackets are. 

Response: the figures in the brackets are 95% confidence interval. The information has been added wherever needed. 

L284- in this experiment you have in effect several parameters that are not constant. You only discuss age of flies but what about their mating status? The youngest age group will not have mated but the other two are likely to have. I know in the discussion you talk about sexual maturity but what about the differences of mating status on susceptibility. Mated and unmated females have different behavioral patterns Ferguson, Calum TJ, et al. "The sexually dimorphic behaviour of adult Drosophila suzukii: elevated female locomotor activity and loss of siesta is a post-mating response." Journal of experimental biology 218.23 (2015): 3855-3861. Is there any evidence that mating status impacts tolerance? 

Response: this is an interesting remark; we did not observe and measure the mating status of the tested flies. The females over 24 hours old are probably all mated, but the question remains for the females under 24 hours old. In Ferguson, Calum TJ, et al. (2015), the tested D. suzukii females are under 4 hours old post hatching in order to ensure their virginity. Odeen et al. (2008) tested that females D. melanogaster of 10-12 hours old did not produce larvae whereas females of 22-25 hours old completed reproduction. Colinet et al. (2016) based their testing on the same estimation: they considered D. melanogaster adult females as virgin under 12 hours old. We guess there is a mix of mated and unmated females within our 0-24h old age class. In our result, females of 0-24h were more susceptible to Phosmet than females of 24-48h and 48-96h old. It would be interesting indeed to test if the mating status affects insecticide tolerance; we added this observation in the discussion (line 475). 

L285- Remove ‘mild but’ from sentence. 

Response: done. 

L334- what active are you talking about? 

Response: the insecticide used was Phosmet, the modification is done. 

L345- again replicates… it would be better to have table S1 in the actual manuscript but make it clearer how many repetitions of each number of flies you have. Do you have 5 replicates of 29 males? It’s just not very clear as it makes me suspicious that there is actually only one replicate of 29. 

Response: this has been answered in more details in the general comment section. There was not indeed 5 replicates of 29 males. We have replaced the term ‘replicate’ that was clearly confusing by bioassay throughout the manuscript. The table S1 have been completed and moved to the ‘Materials and Methods’ section. 

L395- You don’t explain in the introduction, method or results that pyrethroids target the sodium channel - this as a key point. Why didn’t you do the experiments with phosmet? You need to make it clearer why you did what you did. Readers who are not aware of MOA’s will not understand why you did this work on this active. 

Response: thank you for this remark. We have mentioned the sodium channel several times, but we agree that it needs to be clarified that it is the target of pyrethroids and the reason we chose it for Experiment 5. Details have been added (lines 129, 167-170 and 218-219 of the unmarked revised version). 

L463- Assessing mortality too early may distort results- also mortality does not always result in population reduction. Look at the paper Shaw, B., et al. (2019). "Implications of sub-lethal rates of insecticides and daily time of application on Drosophila suzukii lifecycle." Crop Protection 121: 182-194. Several elements are relevant to your manuscript. 

Response: thank you for pointing out this reference. A sentence and the reference was added to the discussion (lines 474-475 of the unmarked revised version).

Attachment Submitted filename: Response to Reviewers.docx Click here for additional data file.

# Peer review: Decision Letter 1

**DOI:** 10.1371/journal.pone.0247756.r003

**Authors:** Maohua Chen

15 Feb 2021

How varying parameters impact insecticide resistance bioassay: an example on the worldwide invasive pest Drosophila suzukii

PONE-D-20-13087R1

Dear Dr. Barrès,

We’re pleased to inform you that your manuscript has been judged scientifically suitable for publication and will be formally accepted for publication once it meets all outstanding technical requirements.

Within one week, you’ll receive an e-mail detailing the required amendments. When these have been addressed, you’ll receive a formal acceptance letter and your manuscript will be scheduled for publication.

An invoice for payment will follow shortly after the formal acceptance. To ensure an efficient process, please log into Editorial Manager at http://www.editorialmanager.com/pone/, click the 'Update My Information' link at the top of the page, and double check that your user information is up-to-date. If you have any billing related questions, please contact our Author Billing department directly at authorbilling@plos.org.

If your institution or institutions have a press office, please notify them about your upcoming paper to help maximize its impact. If they’ll be preparing press materials, please inform our press team as soon as possible -- no later than 48 hours after receiving the formal acceptance. Your manuscript will remain under strict press embargo until 2 pm Eastern Time on the date of publication. For more information, please contact onepress@plos.org.

Kind regards,

Maohua Chen, Ph.D

Academic Editor

PLOS ONE

Additional Editor Comments (optional):

Reviewers' comments:

Reviewer's Responses to Questions

**Comments to the Author**

1. If the authors have adequately addressed your comments raised in a previous round of review and you feel that this manuscript is now acceptable for publication, you may indicate that here to bypass the “Comments to the Author” section, enter your conflict of interest statement in the “Confidential to Editor” section, and submit your "Accept" recommendation.

Reviewer #1: All comments have been addressed

**********

2. Is the manuscript technically sound, and do the data support the conclusions?

The manuscript must describe a technically sound piece of scientific research with data that supports the conclusions. Experiments must have been conducted rigorously, with appropriate controls, replication, and sample sizes. The conclusions must be drawn appropriately based on the data presented. 

Reviewer #1: Yes

**********

3. Has the statistical analysis been performed appropriately and rigorously? 

Reviewer #1: Yes

**********

4. Have the authors made all data underlying the findings in their manuscript fully available?

The PLOS Data policy requires authors to make all data underlying the findings described in their manuscript fully available without restriction, with rare exception (please refer to the Data Availability Statement in the manuscript PDF file). The data should be provided as part of the manuscript or its supporting information, or deposited to a public repository. For example, in addition to summary statistics, the data points behind means, medians and variance measures should be available. If there are restrictions on publicly sharing data—e.g. participant privacy or use of data from a third party—those must be specified.

Reviewer #1: Yes

**********

5. Is the manuscript presented in an intelligible fashion and written in standard English?

PLOS ONE does not copyedit accepted manuscripts, so the language in submitted articles must be clear, correct, and unambiguous. Any typographical or grammatical errors should be corrected at revision, so please note any specific errors here.

Reviewer #1: Yes

**********

6. Review Comments to the Author

Please use the space provided to explain your answers to the questions above. You may also include additional comments for the author, including concerns about dual publication, research ethics, or publication ethics. (Please upload your review as an attachment if it exceeds 20,000 characters)

Reviewer #1: Thank you for addressing the issues raised in the first review and verifying points that needed additional clarification. I am happy with the corrections and appreciate you taking point on board. It reads much clearer now and I think the results are easier to interpret.

**********

7. PLOS authors have the option to publish the peer review history of their article (what does this mean?). If published, this will include your full peer review and any attached files.

If you choose “no”, your identity will remain anonymous but your review may still be made public.

**Do you want your identity to be public for this peer review?** For information about this choice, including consent withdrawal, please see our Privacy Policy.

Reviewer #1: No

# Editor report: Acceptance letter

**DOI:** 10.1371/journal.pone.0247756.r004

**Authors:** Maohua Chen

22 Feb 2021

PONE-D-20-13087R1 

How varying parameters impact insecticide resistance bioassay: an example on the worldwide invasive pest *Drosophila suzukii*

Dear Dr. Barrès:

I'm pleased to inform you that your manuscript has been deemed suitable for publication in PLOS ONE. Congratulations! Your manuscript is now with our production department. 

If your institution or institutions have a press office, please let them know about your upcoming paper now to help maximize its impact. If they'll be preparing press materials, please inform our press team within the next 48 hours. Your manuscript will remain under strict press embargo until 2 pm Eastern Time on the date of publication. For more information please contact onepress@plos.org.

If we can help with anything else, please email us at plosone@plos.org. 

Thank you for submitting your work to PLOS ONE and supporting open access. 

Kind regards, 

PLOS ONE Editorial Office Staff

on behalf of

Prof. Maohua Chen 

Academic Editor

PLOS ONE
